# Isolation of circulating tumor cells in patients undergoing surgery for esophageal cancer and a specific confirmation method

**DOI:** 10.3892/ol.2019.10878

**Published:** 2019-09-18

**Authors:** Yunpeng Zhao, Shukang Zhao, Yingjie Chen, Xiaopeng Dong, Chuanliang Peng, Qifeng Sun, Lei Shan, Zhendan Wang, Xiaogang Zhao

Oncol Lett 17: 3817-3825, 2019; DOI: 10.3892/ol.2019.10017

Subsequently to the publication of this article, the Editor of *Oncology Letters* received notification that the use of the abbreviation “ISET” in this paper represented an infringement of a trademark owned by the company “Rarecells”, who are the exclusive licensee of several patents covering the “ISET” system and of the ISET^®^ device. Upon drawing this matter to the authors' attention, they noted that their use of the term “ISET” as an abbreviation was solely to represent the technique of “isolation by size of epithelial tumor cells”. Nevertheless, so as not to cause any confusion, the authors have agreed to the removal of this abbreviation from their paper. Therefore, the term “ISET” should not have been used in the following nine instances in this article: p. 3817, the Abstract, lines 11 and 19; the Introduction, lines 26 and 31; p. 3818, the subheading “*ISET assay*”; and p. 3822, the Discussion, lines 10 and 11, and p. 3824, the second paragraph, lines 6 and 8. Note that the online version of the paper has been amended to reflect these changes.

The authors regret any inconvenience caused in this regard. Note that these changes regarding the removal of the abbreviation “ISET” from this article do not affect any of the results or conclusions reported in this study.

